# Farewell

**DOI:** 10.3325/cmj.2011.52.651

**Published:** 2011-10

**Authors:** Nika Matovac, Biljana Andrijević-Derk

Dear readers,

October is, as you may know, the month dedicated to the fight against breast cancer and therefore my aunt, Biljana Andrijević-Derk, wanted to share her experience of her sister’s fight with that heavy disease…

“Time heals everything”…is just a phrase. Unfortunately, time does not heal everything, it only underlines the reality, sorrow, and void left behind your lost loved ones. With time we just learn how to cope with this relentless reality.

There is no difference between saying goodbye or see you later, because the content of what you say, and not the form, is what counts. One should be able to say goodbye to the people one loves. This is the final goodbye to a younger “older” sister who never stopped backing me up. I do not know whether to start from the beginning or the end, but the beginning and end have their meeting point and form a circle that we so pompously call life.

It is not easy to continue writing where VESNA left off. Being Vesna’s sister was also not easy, although it was nice. Also, it is not easy being a doctor who is there when the things get tough and at the same time suppresses a scream in the face of the inevitable related to someone who is not your patient, and yet she is.

I will start from the end, when the time and the gravity of the situation did not leave room for saying goodbye to VESNA. I did not find the time, and she did not want to hear, because she did not want to leave.

Having in your family a young and courageous fighter, who had been fighting the uninvited guest as a lion for years, is a hard thing to deal with. This is hard when you are a layman, and it is even harder when you are a doctor. Because, despite the fact that everything has to be told to the patient, and Vesna always wanted to know the hard truth, and was curious and informed nearly as an oncology resident, a small part of truth always remains hidden, the truth you are aware of as a doctor, but as a sister you keep buried deep inside you. This is where “evidence based” medicine ends, and sisterly love and connection begins.

## October 26, 2009

On an October evening, after many years of Vesna’s treatment but much before she left, I had received a “legacy” e-mail:

”My dearest and only sister, this is not a day to remember. I keep crying because of dad’s face when I came home today … You just cannot imagine how sad he looked … Please, do not let all this make you sad, I know that you will be responsible to take care of them when they get old, and I am counting on you to be a part of Nika's life. This does not mean that I am preparing to die, this year I am just processing the fact that I have a metastatic disease for which science knows the final outcome. I am trying very hard with the help from all of you to hold things together for as long as possible …

Vesna, your younger sister, the one you have never stopped backing up …”

This e-mail, which I could not show to our parents until she had left us, shows that all patients have an intuition of their own, but do not want to admit some things publicly, to verbalize them… Some cannot talk about anything else, some are in denial, but all still have hope, no matter how small their chances are. It is precisely because of that HOPE not everything has to be said, something has to be preserved only for the “white caste,” as Vesna angrily used to call the doctors she met during her treatment. During her bilious rants, which would sometimes continue on and on, my doctor’s pride would be hurt, but at that moment I stopped being a doctor, and became only the sister.

## June 2010, Munich

We are leaving for Munich to go through the pleura radio-tomotherapy together once again. This is a somewhat pioneering and experimental method, since it is available in only 2 centers in Germany. Exhaustion, sorrow, and fears are her faithful companions, and the fear and waking up at night in order to check if she is still breathing is my everyday reality.

Youtube is playing Balašević, Bešlić, Gubec Quartet, and everything you cannot forget from the days of youth, and we keep on singing our hearts out just like we used to do before, disregarding the silence of the hotel rooms and the deafening noise of overflowing emotions.

“We could’ve done it all if the day had been longer, if only you’d found a little time for me,” Jadranka Stojaković is singing from a long time ago, now in Japan, while we are in Munich listening to one of her favorite songs, just like Gloria Gaynor’s “I will survive.”

## February 2011

They say that there’s no sorrow like parents’ sorrow. This is true, but it is equally devastating when you realize that you will be left alone … alone with the “legacy” e-mail, without saying anything for goodbye.

She did not say anything because her still alive hopes did not allow her to hear the words of goodbye. Precisely because of that hope, during the sleepless hospital nights, gasping for air, leaning on each other and hugging, two sisters were silent and building sand castles, traveling to Prof. Klepetko to receive a glimmer of light from Bethlehem, and they didn’t want to say anything until the end.

She hoped even when she could reach for a glass of water with her last strength. She hoped when they told her that there was no operation for her. She hoped even when she was not able to say “thank you.” Not even once in those 9 and a half years of treatment did she utter the words “thank you” because for sisters such things go without saying. THANK YOU meant her goodbye, her farewell, her see you later.

And this column is just a small part of what sisters went through together during the treatment. This is also my farewell and a see you later to VESNA, who was not only my sister, but my hope, and also your light of Bethlehem.

Vesna Andrijević Matovac – born on Christmas, died on Valentine’s day...

